# Comparative Analysis of Fetal Kidney Length, Descending Colon Diameter, and Arterial Indices in Intrauterine Growth Restriction Versus Normal Growth Fetuses: A Case-control Study: Fetal Kidney Length, Colon Diameter, and Arterial Indices in Growth-Restricted vs. Normal Fetuses

**DOI:** 10.31661/gmj.v14i.3641

**Published:** 2025-04-16

**Authors:** Haniye Abdolrazagh Nejad, Mahyar Mohammadifard, Alireza Mirgholami, Bita Bijary

**Affiliations:** ^1^ Department of Radiology, Imam Reza Hospital, Birjand University of Medical Sciences, Birjand, Iran; ^2^ Cardiovascular Disease's Research Center, Birjand University Medical of Sciences, Birjand, Iran

**Keywords:** Intrauterine Growth Restriction, IUGR, Kidney Length, Descending Colon Diameter, Doppler Ultrasound, Resistance Index, Pulsatility Index

## Abstract

**Background:**

Intrauterine growth restriction (IUGR) refers to poor fetal growth characterized by a multitude of neonatal complications, necessitating timely diagnosis and intervention. This study aimed to compare the descending colon diameter and kidney length between fetuses with IUGR and normal growth.

**Materials and Methods:**

In this case-control study, a total of 60 participants, 30 pregnant women with IUGR fetuses and 60 women with normal fetuses, referring to an institutional tertiary hospital in eastern Iran, in 2023, were surveyed. Variables included demographic data, fetal kidney length, descending colon diameter, and the pulsatility index (PI) and resistance index (RI) of maternal and fetal arteries. Statistical analysis was performed using IBM SPSS 18.0, with a significance threshold of P<0.05.

**Results:**

A total of 90 pregnant women with a mean age of 29.50 ± 6.61 years were included. The prevalence of patients with occupational employment was significantly higher in the IUGR group (P=0.012). The mean right and left kidney lengths and the anteroposterior diameter of the renal pelvis were significantly higher in normal fetuses compared to those with IUGR (P<0.05). However, no significant difference was found in descending colon diameter between the two groups (P=0.071). The RI and PI of the umbilical and uterine arteries were significantly higher in IUGR fetuses, while the RI and PI of MCA were higher in normal fetuses (P<0.05).

**Conclusion:**

Descending colon diameter is not a reliable marker for IUGR diagnosis. However, the use of RI and PI of umbilical, uterine, and cerebral arteries can be effective in identifying IUGR.

## Introduction

Intrauterine growth restriction (IUGR) is a critical condition in obstetrics, defined by the failure of a fetus to achieve the expected growth during pregnancy. Affecting approximately 5-10% of all pregnancies globally, IUGR is associated with increased susceptibility to perinatal morbidity and mortality, as well as long-term developmental challenges for the child [1]. The condition may arise due to a multitude of maternal, fetal, and placental factors that impair adequate nutrient and oxygen accessibility. Timely detection and management of IUGR are essential for improving outcomes. Nevertheless, achieving accurate diagnosis still remains a major challenge in obstetric care [2].

One of the complexities in managing IUGR stems from the difficulty in differentiating between fetuses that are constitutionally small and those that are pathologically growth-restricted. While small-for-gestational-age (SGA) fetuses may not develop adverse outcomes, IUGR fetuses are at risk of developing complications such as preterm birth, stillbirth, and admission to neonatal intensive care unit (NICU) [1][2]. This emphasizes the importance of identifying reliable diagnostic markers that can distinguish between normal and pathological growth patterns. Currently, IUGR is often diagnosed using a combination of fetal biometry, Doppler ultrasound of fetal and maternal arteries, and monitoring of fetal movements. However, these methods are limited in terms of sensitivity, specificity and accuracy, necessitating further research for identifying additional markers or repurposing already established indicators that may contribute to precise diagnosis [3]. Fetal kidney length and descending colon diameter have recently garnered attention as potential indicators of fetal growth abnormalities. The kidneys, as vital organs responsible for amniotic fluid production and other metabolic functions, may exhibit developmental abnormalities in cases of IUGR [4]. Studies have suggested that impaired placental function in IUGR can affect kidney growth, leading to smaller-than-expected kidney size [5]. Measuring kidney length via ultrasound could thus offer insight into the extent of fetal growth restriction, especially when other conventionally used markers provide ambiguous or borderline results.

Similarly, the descending colon diameter is another anatomical feature that could reflect the fetal growth status. This is because the development of the gastrointestinal tract, including the colon, is influenced by the fetal nutritional and oxygenation status. Recent investigations have provided a linear inverse correlation between colon diameter and gestational age [6][7], which can be translated into prenatal care in the case of IUGR, as well. Theoretically, restricted blood flow and nutrient supply in IUGR might result in a smaller descending colon diameter compared to fetuses with normal growth [8][9]. However, the clinical utility of this measurement remains debated, as research on its effectiveness as a diagnostic tool for IUGR has yielded mixed results.

In addition to these anatomical markers, the use of Doppler ultrasound to assess arterial indices, particularly the pulsatility index (PI) and resistance index (RI) in key fetal and maternal arteries, has become a cornerstone in the evaluation of IUGR [10]. Implement routine Doppler ultrasound assessments in high-risk pregnancies, particularly for women with conditions such as hypertension, diabetes, or a history of IUGR. Recommend regular follow-up scans for patients identified as at risk for IUGR, using Doppler ultrasound to monitor blood flow in the umbilical artery and other relevant vessels. These indices reflect the resistance to blood flow within the uteroplacental and fetal circulations. Elevated PI and RI values in the umbilical and uterine arteries have been linked to poor placental function, a characteristic feature of IUGR [11]. Alternatively, reduced PI and RI in the fetal middle cerebral artery (MCA) often indicate brain-sparing effects, a compensatory mechanism where blood is preferentially directed to the fetal brain, while sparing other organs [12][13]. These Doppler indices provide valuable information about the hemodynamic status of the fetus and are widely used in clinical practice to monitor IUGR pregnancies.

Current diagnostic approaches toward IUGR rely on combining multiple measurements and clinical observations. Therefore, exploring new parameters, such as fetal kidney length and descending colon diameter, in conjunction with established Doppler indices, could enhance diagnostic accuracy. This would help clinicians differentiate between IUGR and normal growth more effectively, allowing for earlier intervention and more personalized prenatal care [3].

This study aimed to compare fetal kidney length, descending colon diameter, and arterial Doppler indices in IUGR and normal-growth fetuses. By analyzing these parameters, we sought to contribute to the refinement of diagnostic tools for IUGR, offering potential avenues for more accurate identification and better management of affected pregnancies.

## Materials and Methods

**Table:** Table1. Diagnostic Criteria for Early and Late Fetal Growth Restriction based on Ultrasound Findings

**Early FGR (GA≤32 weeks) **	**Late FGR (GA>32 weeks) **
AC or EFWr<3 percentile **or UA-AEDF**	AC or EFW<3 percentile
**OR**	or 2/3 of the following criteria:
**1. AC or EFW<10 centile and ** **2. UtA-PI>95 centile and/or ** **3. UA-PI>95 centile **	1. AC or EFW<10 centile and 2. AC or EFW drop>2 quartile on the growth chart 3. CPR<5 centile or UA-PI>95 percentile

**AC:** abdominal circumference; **AEDF:** no diastolic flow; **CPR:** cerebroplacental ratio; **EFW:** estimated fetal weight; **GA:** gestational age; **PI:** pulsation indicator; **UA:** umbilical artery; **UtA:** uterine artery

**Table:** Table2. Demographic Information of Patients in the IUGR and Control Groups

**Demographic**		**Patient (N=90) **		**P-value ^†^**
		IUGR (N=30)	Control (N=60)	
**Age**	Mother (year)	30.32 ± 8.31	29.1 ± 5.62	0.532
	Fetus (week)	31.4 ± 3.74	32.03 ± 2.58	0.094
**Fetus Gender **	Female	18 (60%)	29 (48.3%)	0.372
	Male	12 (40%)	31 (51.7%)	
	Illiterate or Elementary School	3 (10%)	9 (15%)	
**Patient Education **	Middle School	3 (10%)	9 (15%)	0.304
	High School/Diploma	17 (56.7%)	21 (35%)	
	College/University	7 (23.3%)	21 (35%)	
**Residence**	Rural	8 (26.7%)	14 (23.3%)	0.797
	Urban	22 (73.3%)	46 (76.7%)	
**Occupation**	Employed	9 (30%)	5 (8.3%)	0.012
	Unemployed	21 (70%)	55 (91.7%)	

†P-values for categorical variables were calculated using the Chi-squared test, while P-values for quantitative variables with normal and non-normal distribution were calculated using the independent sample’s t test and Mann-Whitney U test, respectively

### Study Design

This was a case-control study aimed at comparing fetal kidney length and descending colon diameter between fetuses with intrauterine growth restriction (IUGR) and normal fetuses.

### Study Population

The present investigation included pregnant women referred to the radiology department at an institutional tertiary hospital located in Birjand, Iran, in 2023. The case group consisted of 30 pregnant women diagnosed with IUGR, and the control group included 60 women with normal fetuses. The sample size of 30 IUGR and 60 normal fetuses was determined based on a power analysis using Cochran’s formula, which indicated that this size would provide sufficient power (85%) to detect significant differences in the primary outcomes. To minimize selection bias, we employed strict inclusion and exclusion criteria for participant recruitment. All participants were consecutively enrolled from the radiology department of our institution, ensuring that both groups were comparable.

### Patient Eligibility Criteria

Patient eligibility for the study was determined using a set of predefined inclusion and exclusion criteria. To be included, patients had to have a gestational age between 25 and 37 weeks and a diagnosis of intrauterine growth restriction (IUGR) based on ultrasound and umbilical artery color Doppler criteria. Patients were excluded from the study if they had pregnancies with multiple fetuses, major congenital anomalies, known chronic diseases such as diabetes or hypertension, or if they failed to provide informed written consent.

A set of predefined inclusion and exclusion criteria were used to evaluate the eligibility of patients before enrolling them to the study. Patient inclusion criteria were as follows:

* Gestational age between 25 and 37 weeks

* Diagnosis of IUGR based on ultrasound and umbilical artery color Doppler criteria

Additionally, patients with the following conditions were duly excluded from the study:

* Pregnancies with multiple fetuses

* Major congenital anomalies

* Pregnant women with known chronic diseases (e.g., diabetes, hypertension)

* Failure to provide informed written consent

### IUGR Diagnosis

The diagnostic criteria for Intrauterine growth restriction (IUGR) were applied consistently by trained radiologists using standardized ultrasound protocols[14][15]. IUGR is linked to increased perinatal morbidity and mortality. It is defined as a fetus that does not reach its growth potential. Antenatal small for gestational age (SGA) refers to a fetus with a weight below the 10th percentile[14][16]. IUGR and SGA are often used interchangeably. Identifying IUGR is crucial and starts with evaluating risk factors. The diagnosis is confirmed through ultrasound biometry, which indicates an estimated fetal weight (EFW) of less than the 10th percentile. We ensured that all ultrasound assessments were performed by experienced technicians who followed the established criteria for IUGR diagnosis, including fetal weight percentiles and Doppler assessments. This consistency helps to mitigate discrepancies in diagnosis. As mentioned in the eligibility criteria, diagnosis of IUGR was based on ultrasound and umbilical artery color Doppler criteria, which are summarized in Table-1. The cerebroplacental ratio (CPR) is calculated by dividing the Doppler flow measurement of MCA by that of UA. Although various indices, such as the systolic/diastolic (S/D) ratio, RI, and PI, can be used for this calculation, PI is increasingly being preferred to other indicators.

### Sample Size and Sampling

The sample size (N0) was calculated using Cochran’s equation (Eq. 1) with margin of error (e) of 0.05, confidence level (z) of 85%, and a 12% prevalence (p) of IUGR, as reported by Eslamian et al. (2022) [17]. A total of 90 participants were enrolled, with 30 in the IUGR group and 60 in the normal fetus group.Study Procedure

All potentially eligible participants underwent initial prenatal care, including clinical examination. For a definitive diagnosis of IUGR, fetal weight percentile was calculated, with values below the 10th percentile confirming IUGR. Umbilical artery color Doppler was used for final confirmation. Patients with a definite diagnosis of IUGR were ultimately recruited to the study as the IUGR group.

Transabdominal and color Doppler ultrasound measurements, including descending colon diameter and kidney length, as well as arterial resistance (RI) and pulsatility (PI) indices, were performed using the radiological equipment available within the department. Demographic data included maternal and gestational age, fetal gender, educational and occupational status and location of residence.

### Ethical Considerations

The protocol of this study was approved by Research Ethics Committee (REC) of Birjand University of Medical Sciences (Approval ID: IR.BUMS.REC.1402.316). Informed written consent were obtained from all patients, after the entire procedure of the study was explained to them. Patient credentials and identifying information were kept confidential throughout the study.

### Statistical Analysis

Data were entered into IBM SPSS Statistics for Windows, version 18 (SPSS Inc., Chicago, Ill., USA) for statistical analysis. Descriptive statistics were reported as frequency or mean ± standard deviation (SD). The Kolmogorov-Smirnov test was used to assess the normality of data distribution. Parametric and non-parametric tests, including the independent sample’s t test and the Mann-Whitney U test, were used to compare quantitative variables with normal and non-normal distribution between groups. Additionally, Chi-squared test was used for comparison of categorical variables, with a significance level (P-value) set at 0.05.

## Results

**Table:** Table3. Comparison of Kidney Size and Descending Colon Diameter between the Two Groups

**Size (mm)**		**Patient (N=90) **		**P-value ^†^**
		IUGR (N=30)	Control (N=60)	
**Kidney Length **	Right	30.3 ± 2.9	35.6 ± 4.8	<0.001
	Left	0.86 ± 0.26	0.48 ± 0.07	<0.001
**AP Renal Pelvic Diameter **	Right	0.80 ± 0.17	0.50 ± 0.1	<0.001
	Left	0.69 ± 0.08	0.80 ± 0.08	<0.001
**Descending Colon Diameter **		1.77 ± 0.2	0.97 ± 0.17	<0.001

†P-value was calculated using independent sample’s t test and Mann-Whitney U test for parametric and non-parametric data.

**Table:** Table4. Comparison of Arterial RI and PI between the Two Groups

**Index**	**Artery**	**Patient (N=90) **		**P-value ^†^**
		IUGR (N=30)	Control (N=60)	
	Umbilical	0.84 ± 0.04	0.61 ± 0.07	<0.001
**Resistance**	Right Uterine	0.86 ± 0.26	0.48 ± 0.07	<0.001
	Left Uterine	0.8 ± 0.17	0.5 ± 0.1	<0.001
	MCA	0.69 ± 0.08	0.8 ± 0.08	<0.001
	Umbilical	1.77 ± 0.2	0.97 ± 0.17	<0.001
**Pulsatility**	Right Uterine	1.96 ± 0.55	0.79 ± 0.27	<0.001
	Left Uterine	1.99 ± 0.54	0.91 ± 0.5	<0.001
	MCA	1.43 ± 0.5	1.98 ± 0.48	<0.001

†P-value was calculated using independent sample’s t test and Mann-Whitney U test for parametric and non-parametric data.

A total of 90 pregnant women with a mean age of 29.50 ± 6.61 participated in the study. Table-1 lists the demographic information of participants, including mothers and their fetuses. Of the 90 participants, 30 were categorized as the IUGR group with a mean age of 30.32 ± 8.31, while the remaining 60 participants were categorized as the control group with a mean age of 29.1 ± 5.62. Of the 90 fetuses, 30 were from the IUGR and 60 were from the control group, with mean gestational ages of 31.4 ± 3.74 and 32.03 ± 2.58 weeks, respectively. No statistically significant differences were observed in maternal and gestational ages between the two groups. In terms of fetus gender distribution, the IUGR group showed a relatively higher female to male ratio compared to that of the control group (1.5 vs 0.935), however, the difference was not found to be statistically significant (P=0.372). Similarly, patient education was not found to a significantly influential factor, with different degrees of educations from either group being comparable to each other (P=0.304). The frequency of residence location was highly similar between the two groups, with comparable rates of rural (26.7% vs. 23.3%) and urban residence (73.3% vs. 76.7%). In contrast, the frequency of occupational status was found to be significantly different between the two groups, with IUGR mothers showing considerably higher rates of employment compared to that of normal mothers (30% vs. 8.3%, P<0.05).

Next, we recorded the size of kidney and descending colon between the two groups as determined based on ultrasound evaluation, the results of which are reported in Table-2. As can be seen, significant differences were observed an all anatomic parameters between the IUGR and control groups. On average, the mean pole-to-pole size of right and left kidneys were significantly lower in the IUGR fetuses compared to their normal counterparts (P<0.001), with right and left kidneys being 30.3 ± 2.9 mm and 30.5 ± 3.2 mm in length in the former group. Similarly, the two groups were found to significantly differ from one another in terms anteroposterior (AP) renal pelvic diameter, with IUGR fetuses showing substantially lower mean right and left AP diameters compared to the right (2.2 ± 0.68 vs. 2.61 ± 0.76 mm) and left (2.17 ± 0.65 vs. 2.45 ± 0.45 mm) AP diameter of the control group (P<0.001). Conversely, we did not find a marked difference in the diameter of descending colon between the IUGR and normal fetuses, which was comparably lower in the former group in relation to the latter, albeit not to a significant extent (P=0.071).

Table-3 presents RI and PI values of umbilical, right and left uterine and MCA arteries, as determined during color Doppler evaluation.As can be inferred from Table-4, all indices corresponding to umbilical, right and left uterine and MCA arteries differed significantly between the two groups (P<0.001). While the mean resistance index of all three arteries in the IUGR group were markedly higher to that of the control group, the arterial pulsatility index values were consistently higher in the IUGR group, reflecting the increased resistance indicated by elevated RI values. The highest resistance index in the IUGR group (0.86 ± 0.26) was recorded for the right uterine artery, which corresponded to the lowest resistance index (0.48 ± 0.07) for the same artery in the control group, a difference that was deemed as statistically significant (P<0.001). The highest pulsatility index, on the other hand, was reported for the left uterine artery (1.99 ± 0.54) in the IUGR group, whereas the highest PI value among normal patients was reported for MCA (1.98 ± 0.48).

## Discussion

Based on the findings of the current study, no significant differences were observed in maternal age or fetal gestational age between the IUGR and normal groups, which aligns with the study by Mohamed Abdelghany et al. (2023), who reported similar maternal age distribution between IUGR and normal-weight fetuses [18]. In contrast, we observed significant differences in kidney dimensions, with the mean lengths of both the right and left kidneys, as well as the anteroposterior diameter of the renal pelvis, being greater in normal-weight fetuses compared to those with IUGR. This is consistent with the findings of Silver et al. (2003), who demonstrated that renal volume in IUGR fetuses was significantly reduced compared to normal fetuses, supporting the association between IUGR and diminished kidney development [19]. However, no significant difference in the mean diameter of the descending colon was found between the two groups in our study, which contrasts with the results reported by D’Inca et al. (2011), who suggested that IUGR might be associated with a thinner and longer intestine in animal models. This discrepancy could be attributed to the differences in study design and species, as the latter study was conducted on piglets, which may exhibit different growth patterns compared to human fetuses [20]. Overall, our findings contribute to the growing body of evidence that IUGR significantly affects fetal organ development, particularly the kidneys, with potential long-term implications for health.

The results of the current work showed that the mean RI and PI of the umbilical artery in fetuses with IUGR were markedly higher than in normal fetuses. These findings are in agreement to those reported by Wajid et al., in 2022, who noticed a marked increase in the PI of the umbilical artery (UA) in IUGR fetuses, suggesting a diagnostic accuracy of 89% for UA color Doppler in the clinical diagnosis of IUGR [21]. Additionally, we observed that the mean RI and PI of the right and left uterine arteries were substantially higher in fetuses with IUGR compared with their normal counterparts. In 2020, Adefisan et al. prospectively surveyed 120 pregnant women from Nigeria with gestational ages ranging from 22 to 26, aiming explore the value of second-trimester UA color Doppler ultrasound in determination of pregnancy-related adverse outcomes. They observed no significant difference in the PI of UA between IUGR and normal fetuses, which contrasts with our results [22]. This discrepancy could be attributed to variations in race, gestational age, and the number of pregnancies among the patients. From 2016 to 2024, several studies from different countries reported significantly higher mean PI and RI values of uterine arteries in IUGR fetuses compared to those with normal growth [18][23][24][25], which collectively support the findings reported in this study. In 2023, Mohamed Abdelghany et al. investigated the significance of uterine perfusion in prediction of late onset IUGR in 119 high-risk pregnant women, suggesting a relationship between uterine artery PI and the likelihood of IUGR [18]. In 2023, Gebreil et al. evaluated the relationship between the first-trimester uterine artery Doppler indices and IUGR in a population of 120 pregnant women from Egypt, reporting substantially higher RI and PI values among patients with IUGR compared to controls [25]. An earlier study with a comparable premise, conducted by Drouin et al. in 2018, investigated the applicability of uterine artery PI in the prediction of small-for-gestational age (SGA) among 4,610 participants from Canada, disclosing a positive correlation between uterine artery PI and the likelihood of SGA, highlighting the clinical value of UA color Doppler assessment in timely diagnosis of growth restrictions [24]. Similarly, Abdel Moety et al. concluded that uterine artery PI was a perfectly sensitive (100%) marker for prediction of IUGR [23].

In contrast to the aforementioned studies, Lane et al. found no significant difference in the PI of the uterine artery between IUGR and normal fetuses in a murine model of IUGR [26], which differs from our findings. The primary reason for this discrepancy could be the physiological differences between other mammals and humans. Increased uterine artery blood flow in hypoxic conditions is an adaptive mechanism aimed at compensating for reduced oxygen, but this is insufficient to prevent IUGR. During a normal pregnancy, uterine artery blood flow increases by at least 20-fold, facilitating adequate supply of nutrients and oxygen to the growing fetus [27].

Our study found that the mean RI and PI of the MCA were significantly lower in fetuses with IUGR compared to those with normal growth, which is consistent with previous findings. Mu et al. reported that the PI of the MCA was notably reduced in IUGR fetuses. This reduction in PI was linked to fewer detectable intraplacental villous arteries, highlighting compromised blood flow in IUGR cases [28]. Similarly, Abel et al. observed lower PI values in IUGR fetuses, emphasizing the role of MCA Doppler measurements in assessing fetal well-being [29]. In 2022, Coenen et al. also demonstrated that altered Doppler parameters, including PI, were associated with adverse outcomes in IUGR, further supporting its clinical relevance [30]. Other studies confirmed that abnormal MCA Doppler indices, particularly elevated RI and PI, are strong predictors of IUGR, providing valuable insight for early detection and management [31][32][33][34][35][36][37].

Overall, In conclusion, our findings align with those of Fardiazar et al., who demonstrated that Doppler assessments of the umbilical, uterine arteries, and fetal MCA are effective in detecting IUGR [38]. Additionally, Alimohammadi et al. highlighted the role of increased systolic/diastolic index in identifying IUGR, emphasizing on the importance of Doppler ultrasonography in clinical practice [39]. Moreover, Karakus et al. further supported this by showing that altered aortic isthmus Doppler measurements were associated with adverse neonatal outcomes in IUGR cases [40][41]. Taken together, these studies emphasize the critical role of Doppler ultrasound in monitoring blood flow changes and placental insufficiency, which become increasingly evident as pregnancy progresses in IUGR cases. This highlights the necessity of integrating comprehensive Doppler evaluations in the management of pregnancies at risk of IUGR for timely interventions.

## Conclusion

The authors aim to enhance the understanding and detection of intrauterine growth restriction (IUGR) to improve outcomes for affected pregnancies and infants. The study found that Doppler ultrasonography of the umbilical artery, uterine arteries, and fetal middle cerebral artery (MCA) provided valuable information for detecting IUGR. Significant changes in kidney dimensions and Doppler indices (resistive index and pulsatility index) were observed in IUGR fetuses, indicating reduced placental blood flow and organ underdevelopment. Increased resistance in the umbilical and uterine arteries, along with reduced MCA Doppler indices, were noted in IUGR cases, supporting the effectiveness of Doppler ultrasound in assessing blood flow and placental insufficiency for timely diagnosis and intervention. However, the study had limitations, including a small sample size of 30 IUGR and 60 normal fetuses, potential variability in ultrasound measurements due to differences in operator skill, and inadequate control of factors influencing fetal growth. The case-control design may limit the generalizability of the findings, and the study’s single-institution setting may affect the diversity of the sample. Further research with larger and more diverse cohorts is needed to validate these findings and explore long-term outcomes associated with IUGR.

## Conflict of Interest

None.
